# Fire Growth Behavior and Predictive Modeling of 3D-Printed Triply Periodic Minimal Surface (TPMS) Porous PLA Structures

**DOI:** 10.3390/polym18141788

**Published:** 2026-07-22

**Authors:** Mingyang Guo, Yachao Wang, Dongzhao Lu, Henri Vahabi

**Affiliations:** 1 State Key Laboratory of Tropic Ocean Engineering Materials and Materials Evaluation, Haikou 570228, China; 2 School of Resources Engineering, Xi’an University of Architecture & Technology, Xi’an 710055, China; 3 National & Local Joint Engineering Research Center of Technical Fiber Composites for Safety and Health, School of Textile and Clothing, Nantong University, Nantong 226019, China; 4 State Key Laboratory of Safety and Resilience of Civil Engineering in Mountain Area, Nanchang 330013, China; 5 LMOPS, CentraleSupélec, Université de Lorraine, 57000 Metz, France

**Keywords:** triply periodic minimal surface, 3D printing, regression model, fire growth, polylactic acid

## Abstract

Triply periodic minimal surfaces (TPMSs) are increasingly used in thermal management systems due to their high surface area and interconnected porous architecture. However, how TPMS geometry influences fire growth remains poorly understood. Herein, Gyroid, Diamond, and Schwarz-P PLA structures were fabricated through additive manufacturing, and their fire behavior was characterized via cone calorimetry. Univariate experiments demonstrate that flame propagation is primarily governed by cell structure, rather than porosity or specific surface area. The orthogonal array L_9_(3^3^), covering three factors of wall thickness, cell size and cell type (Gyroid, Diamond, and Schwarz-P), indicates that their effects on the flame growth index (FGI) rank as: cell size > unit cell > thickness. Meanwhile, a multivariate regression model was developed to predict the FGI of TPMS porous PLA, exhibiting high prediction accuracy (R^2^ = 0.95) and low residual standard deviation. These results provide quantitative insights into the correlation between TPMS geometric features and fire growth, which facilitates the design of safer porous structures for thermal management applications.

## 1. Introduction

Triple-period minimal surfaces (TPMSs) are characterized by highly ordered, interconnected porous structures with high specific surface area, and periodic implicit surfaces with zero mean curvature [1,2]. Combined with the ease of fabrication by additive manufacturing and the favorable processability of polylactic acid (PLA) [3,4], TPMS structures have attracted considerable attention in advanced engineering applications [5,6]. Their highly interconnected pore network can be tailored to optimize fluid flow and heat transfer in heat exchangers, heat sinks, and other thermal management components through 3D printing [7,8,9]. Moreover, additive manufacturing gives the possibility for a precise fabrication of complex TPMS geometries with excellent dimensional accuracy and structural integrity.

TPMS porous materials fabricated by additive manufacturing methods have attracted considerable attention [10,11] as thermal management material [12] due to their excellent mechanical and thermal performance [13]. Their highly interconnected porous architecture can disrupt fluid boundary layers and promote convective heat transfer [14]. The highly interconnected through-hole structure is more conducive to fluid flow and forced convection heat dissipation, which could be used to design compact heat exchangers, but different pore sizes affect its heat transfer [15,16]. Heat transfer characteristics play a crucial role in material heating and combustion processes. Therefore, it is important to understand how TPMS geometry influences fire development to design such structures.

Meanwhile, quantitative research on the heat convection of porous polylactic acid (PLA) usually requires three dimensions: experimental measurement, numerical simulation, and theoretical analysis. This involves establishing a quantitative relationship between pore architecture, flow parameters, and heat transfer performance, demonstrating that pore size and structure affect heat transfer performance [17,18]. Numerous investigations have examined the heat transfer characteristics of TPMS structures and their potential for efficient thermal management in high heat flux applications [19].Tang et al. [20] assessed the convective heat transfer performance of Gyroid, Diamond, and I-WP TPMS frameworks through experiments and simulations, showing superior heat dissipation compared with conventional designs. Gao et al. [21] noted that the heat transfer rate of TPMS increased with the flow rate, although the efficiency was compromised when employing the laser powder bed technique. Meanwhile, various researchers have examined the mechanical properties of different TPMSs [22], including Poisson’s ratio [23], anisotropy [24], elastic behavior [25], and yield strength [26]. Beyond these individual properties, architected metamaterials exhibit rich multifunctional design potential. For instance, direction-dependent stiffness can be deliberately engineered through topology optimization and validated via combined simulation and experimentation [27]; origami-inspired designs have achieved distinct mechanical responses—with stiffness variations up to threefold—along three orthogonal axes [28]. Such mechanical tunability, together with the tailored thermal transport discussed above, underscores the versatility of TPMS architectures for advanced engineering systems. Since heat transfer plays a critical role in material heating and fire development, variations in TPMS geometry may also influence flame propagation and fire growth. Despite the growing research interest in the thermal and mechanical properties of TPMS, few studies have explored its combustion-related behaviors. Prieto et al. conducted CFD simulations to analyze the thermal–hydraulic characteristics of Gyroid TPMS, but their work focused only on fluid convection performance and did not include combustion behaviors [29]. Existing TPMS combustion simulations remain limited to gas-phase methane combustion in inorganic porous media, and these studies fail to quantitatively characterize the flame spread and fire growth of solid polymer materials [30,31]. Effective strategies for regulating the thermal decomposition and combustion performance of PLA have been widely reported, yet most rely on filler modification or conventional porous structures rather than TPMS topological design [32]. The quantitative correlation between TPMS architectural characteristics and polymer fire growth thus remains poorly understood. However, our previous work systematically investigated the combustion performance of PLA with regular pore structures [33]; we have not yet established the quantitative relationship between TPMS geometric features and fire growth evolution.

Consequently, to clarify the relationship between TPMS architecture and fire growth, three porous PLA structures with different TPMS geometries (Gyroid (G), Diamond (D), and Schwarz-P (S-P)) were fabricated through 3D printing and their combustion behavior was systematically investigated. An orthogonal experimental design was employed to investigate the effects of the TPMS structure’s unit cell, wall thickness, and cell size on the flame growth index (FGI). Furthermore, multiple regression analysis was used to establish a quantitative relationship between FGI and the structural parameters, including TPMS type, cell size and wall thickness. The results provide new insights into the influence of TPMS geometry on fire development and provide useful guidance for the design of TPMS-based thermal management materials.

## 2. Experiment and Methods

### 2.1. Materials

Polylactic acid (PLA) filaments were purchased from Beijing Hongrui Huitianwei Co., Ltd., Beijing, China. The filaments had a commercial standard diameter of 1.75 mm, a melt flow index of 6–8 g/10 min (190 °C/2.16 kg), a tensile strength of 250 kg/cm^2^, a heat deflection temperature of 58 °C, a melting point of 160–165 °C, and a solid density of 1.26 g/cm^3^.

### 2.2. Fabrication of TPMS Structures

**Figure 1:**
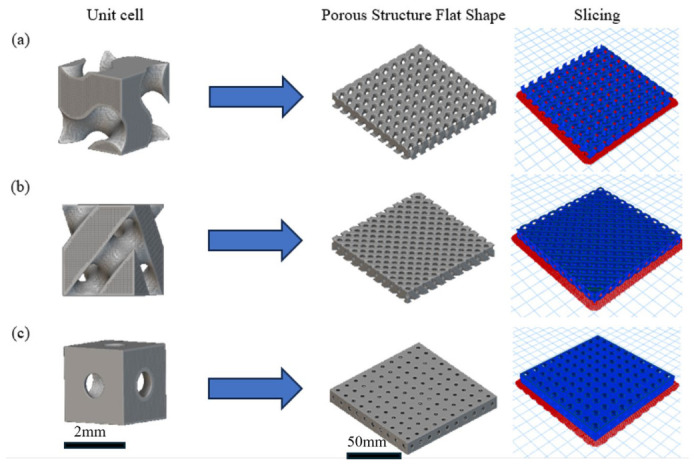
The unit cell of the three TPMSs.

All TPMS porous specimens were fabricated via fused deposition modeling (FDM) using a 3D printer (HORIZ400, Hangzhou, China). The CAD models of Gyroid, Diamond, and Schwarz-P unit cells were created using Ntopology software (Ntopology, New York, NY, USA, v5.22.2), with cell size, wall thickness, and unit cell type as the design variables. The modeling-to-slicing workflow of the three cellular architectures is shown in Figure 1. The models were exported in STL format and subsequently imported into Modelight (Modelight, Shanghai, China, v3.2.0.9) slicing software to generate G-code instructions for printing. Prior to printing, the build platform was coated with adhesive to ensure proper first layer adhesion. The printing parameters were set as follows: nozzle temperature 210–220 °C, bed temperature 40 °C, printing speed 40 mm/s, layer height 0.08 mm, and nozzle diameter 0.4 mm. All samples were printed with 100% infill density to ensure structural integrity and avoid internal voids unrelated to the designed TPMS porosity. The implicit surface equations defining the three TPMS topologies are given in Equations (1)–(4) [34,35]. These architectures are bi-continuous, highly interconnected pore networks, characterized by high specific surface areas and tunable tortuosity.

(1) $$\Phi_{G}=\cos(x)\cdot \sin(y)+\cos(y)\cdot \sin(z)+\cos(z)\cdot \sin(x)=c$$

(2) $$\Phi_{D}=\sin(x)\cdot \sin(y)\cdot \sin(z)+\sin(x)\cdot \cos(y)\cdot \cos(z)+\cos(x)\cdot \sin(y)\cdot \cos(z)+\cos(x)\cdot \cos(y)\cdot \sin(z)=c$$

(3) $$\Phi_{S-P}=\cos(x)+\cos(y)+\cos(z)=c$$

(4) $$\Phi =\{(x,y,z)\in R^{3}:f(x,y,z)\geq C\}(or\{(x,y,z):f(x,y,z)\leq C\}$$

where x = 2πX/L_x_, y = 2πY/L_y_, z = 2πZ/L_z_, L_x_, L_y,_ and L_z_ are the unit cell size in X, Y, and Z directions, respectively.

### 2.3. Fire Growth Test

**Figure 2:**
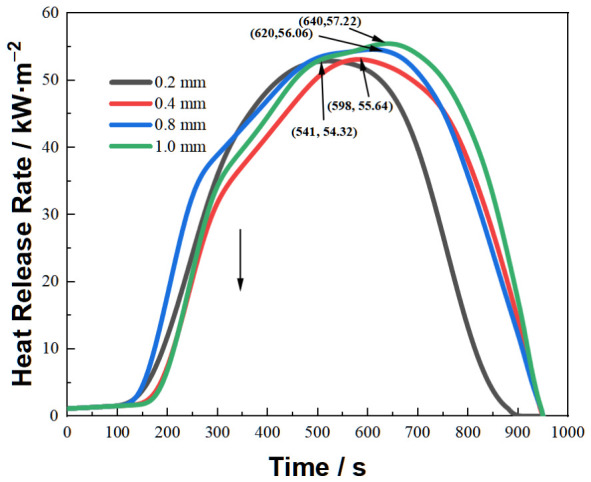
FGI of D−type TPMS porous PLA with different thicknesses.

**Figure 3:**
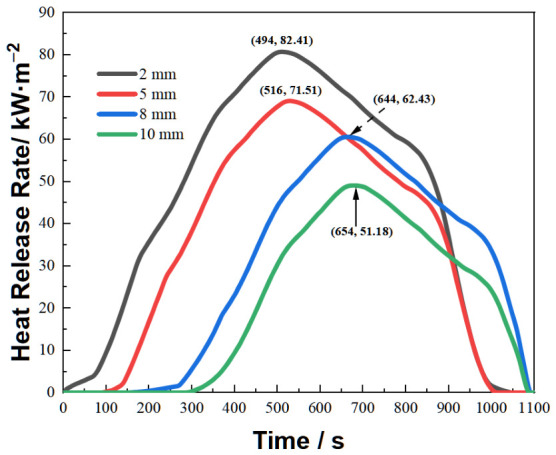
FGI of D−type TPMS porous PLA with different cell sizes.

**Figure 4:**
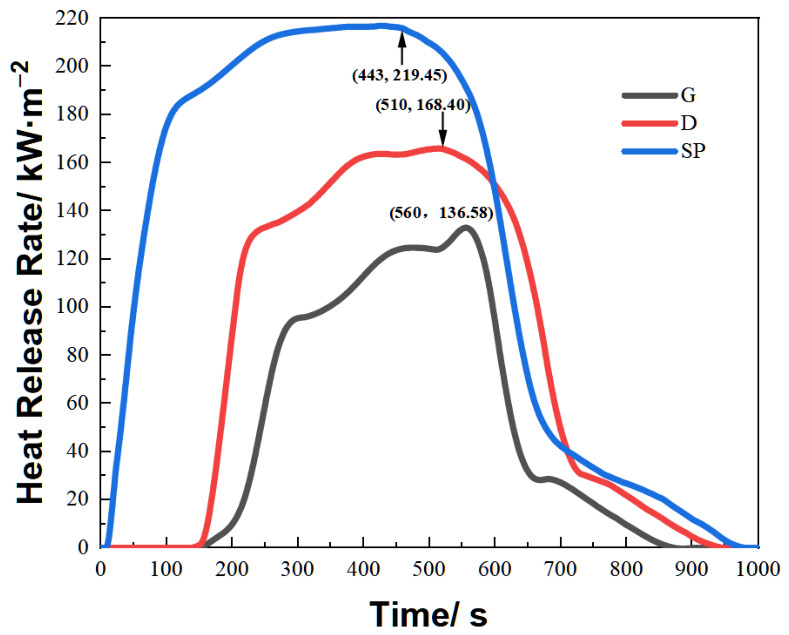
FGI of TPMS porous PLA with different cell types.

**Figure 5:**
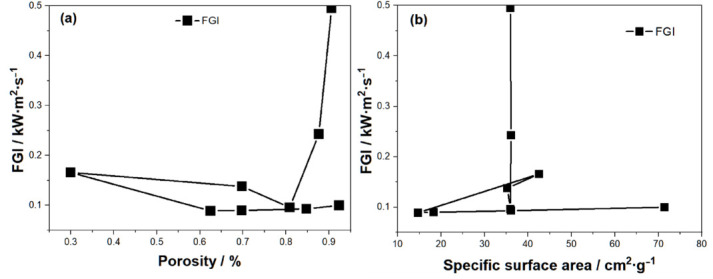
The relationships between FGI and (**a**) porosity or (**b**)specific surface area.

Using a conical calorimeter (CC, ZY6243) from China Zhongnuo Instrument Company (Xinxiang, China), in line with BS ISO 5660-1:2015 [36], the fire growth of the samples was conducted. Samples measuring 100 × 100 × 10 mm^3^ were encased in aluminum foil and set horizontally beneath external radiation of 35 kW·m^−2^ to simulate fire conditions with a distance of 25 mm. The observation period was set at 1040 s, coinciding with the complete combustion time of the PLA. The heat release rate of the samples was recorded in real time, and the peak to heat release rate (pHRR) was obtained to evaluate its fire growth. To ensure reliability, each test was performed three times to achieve reproducibility within 10% for pHRR, the average values were then derived for comparative analysis. All data plotted in Figure 2, Figure 3, Figure 4 and Figure 5 are these triplicate-averaged results, and all replicate deviations satisfy the 10% repeatability criterion of BS ISO 5660-1:2015. The sample mass normalization was applied to eliminate mass effects, ensuring that tested fire growth is expressed per unit mass. The flame growth index (FGI, kW·m^−2^·s^−1^) was calculated using Equation (5), where a lower FGI value suggests a slower fire growth:

(5) $$FGI=\frac{pHRR}{T_{P}}$$

where Tp is the time to pHRR (s). To eliminate the effect of sample mass variation caused by different wall thicknesses and cell sizes, the measured pHRR values were normalized by the initial mass of each specimen before FGI calculation. All reported FGI values are therefore expressed on a per-unit-mass basis.

### 2.4. Structural Characterization

The porosity and volumetric specific surface area of all TPMS specimens were numerically calculated from the CAD models using AreanTopology software (version 5.22.2). The calculations were performed on STL models with a voxel resolution of 0.05 mm, based on the solid PLA density of 1.26 g/cm^3^. Table 4 summarizes the calculated porosity and specific surface area for all samples. These values serve as baseline structural descriptors for the subsequent fire growth analysis, while their correlations with FGI are discussed in Section 3.

### 2.5. Experimental Design and Statistical Analysis

#### 2.5.1. L9(3^3^) Orthogonal Experimental Design

This design approach examines how the thickness (Factor A), cell size (Factor B), and unit cell (Factor C) impact the FGI. Interaction between level factors was excluded according to the orthogonal test L9(3^3^). Three influential factors were identified: thickness, cell size, and unit cell, each with three levels. The 9 groups of samples with various TPMS structures were selected based on the fused 3D printer system’s capabilities. The combinations of each test were listed according to the orthogonal experimental design table. The L9(3^3^) orthogonal experimental tables are shown in Table 1 and Table 2.

For the L9(3^3^) orthogonal experiment, all nine structural combinations were independently fabricated and tested in triplicate, producing 27 individual FGI measurement points for subsequent regression modeling. Each of the nine combinations was fabricated and tested in triplicate independently (three specimens per condition), yielding a total of 27 experimental observations. The averaged FGI of triplicates was used for range analysis and ANOVA, while all 27 individual raw data points were adopted for multivariate regression modeling to fully reflect experimental variability and enhance statistical reliability.

It should be noted that tortuosity, connectivity index and Gaussian curvature govern porous heat and mass transfer physically. However, in our L9(3^3^) orthogonal design, these topological parameters are uniquely determined by cell type, leading to complete multicollinearity. Adding them as continuous regression variables would produce undefined VIF values and distort coefficient fitting. Thus, all topological effects are integrated into the categorical cell type variable, so this work focuses on three adjustable engineering parameters: wall thickness, cell size and unit cell.

#### 2.5.2. Univariate Single-Factor Controlled Experiment

Table 3 presents a series of one-way experimental designs aimed at controlling a single variable, denoted as X, within each experimental set. The study examines the individual effect of thickness, cell size, and unit cell on the FGI.

#### 2.5.3. Range Analysis

Extreme variance analysis is a robust approach for interpreting data from orthogonal tests [37]. This method is straightforward and intuitive, aiding in identifying the relative impact of different factors on test outcomes, which helps in deducing the most effective levels and combinations of factors. Greater values of extreme deviation suggest a more significant effect on the test outcomes, determining the best factor levels and their combinations through the deviation evaluation.

#### 2.5.4. Analysis of Variance

Analysis of variance (ANOVA), frequently referred to as the “F-test,” is employed to determine the significance of variations among the averages of multiple samples, distinguishing the impact of the factor from the experimental noise. In the F-test, the F-value symbolizes the ratio of the total squared deviations to the degrees of freedom, offering critical statistical insights [38]. The F-value is calculated as follows in Equations (6)–(11).

(6) $$V^{2}={\sum \left(X-\bar{X}\right)}^{2}$$

(7) f=K−1

(8) $$f_{e}=N-K$$

(9) $$W_{J}=V^{2}/f$$

(10) $$W_{e}={V^{2}}_{e}/f_{e}$$

(11) $$F=W_{J}/W_{e}$$

Herein, V^2^ is the sum of squared variances, X represents the experimental data, and $\bar{X}$ is the total mean. f denotes the degrees of freedom, K is the number of levels for each factor in a column, and *N* is the total number of observations. W_J_ represents the mean square, and W_e_ represents the mean square of the error column. F-values for different significance levels can be found in the F-test critical value table ($F_{\alpha }$). At a particular level of significance (α = 0.05), when the factor degrees of freedom are f = 2 and the error degrees of freedom are f = 20, the corresponding F-value is *F*_0.05_(2,20) = 3.49. The present ANOVA is based on the L9(3^3^) orthogonal design with three factors at three levels. Each of the nine structural combinations was tested in triplicate (*N* = 27), yielding dftotal = 26. Each factor has df = 2, and thus dferror = 26 − 2 − 2 − 2 = 20. This residual error originates from within-group variability among replicates, including both 3D printing dimensional deviations and cone calorimeter measurement fluctuations. The critical F-values, F_0.05_(2,20) = 3.49 and F_0.01_(2,20) = 5.85, confirm the statistical validity of our significance tests. The significance of the model was evaluated by comparing the calculated F-value with the corresponding critical F-value from the F-distribution table. A higher F-value indicates greater statistical significance of the model and reflects a stronger agreement between the predicted and experimental data.

#### 2.5.5. Multivariate Linear Regression Method

Multivariate linear regression was performed via SPSS (Version 26.0) software to establish the quantitative prediction relationship between FGI and three structural parameters (wall thickness, cell size, unit cell type). All 27 raw replicate FGI data were adopted as the modeling dataset with the Enter regression method. Correlation coefficient (R), coefficient of determination (R^2^), RMSE and MAE were used to evaluate model fitting performance. Tolerance and VIF values were calculated to diagnose multicollinearity, and residual normality/homoscedasticity were verified for model reliability.

## 3. Results and Discussion

### 3.1. Univariate Experiment of TPMS Porous PLA

#### 3.1.1. Effect of Thickness on FGI

Figure 2 depicts the variation in the FGI of TPMS porous PLA with the 10 mm D-type relative to changes in thickness. The pHRR and Tp show positive correlations with the thickness increasing gradually from 0.2 to 1.0 mm; the pHRR of the samples escalates from 54·to 57 kW·m^−2^, an increase of 5.34%; and the TP increased from 541 to 640 s, reflecting an 18.30% rise. However, as the thickness increases, the FGI of the samples decreases from 0.1004 to 0.0894 kW·m^−2^·s^−1^ with a reduction of 10.96%. Because the varied thickness changes the porosity [39], the increased thickness makes the specific surface area and porosity decrease, which is unfavorable for flame propagation accordingly.

#### 3.1.2. Effect of Cell Size on FGI

Figure 3 illustrates the FGI with the cell size increasing from 2 to 10 mm for the D-type samples; the pHRR exhibits a negative correlation, whereas the T_P_ shows a positive correlation with cell size. The pHRR of the samples drops from 82·to 51 kW·m^−2^ with a decrease of 37.9%, while the T_P_ rises from 494 to 654 s with an increase of 32.39%. However, the FGI decreases from 0.1668 to 0.0783 kW·m^−2^·s^−1^ with a reduction of 53.06%. Consequently, an increase in the cell size leads to a significant decrease in the FGI. As the cell size increases, the porosity increases accordingly. However, reducing the cell size increases the frequency of local flow area changes, inducing large-scale bifurcation flow [40], Coupled with intensified flow interactions, this promotes heat transfer and accelerates flame propagation.

#### 3.1.3. Effect of Unit Cell on FGI

Figure 4 depicts the FGI across various unit cells, the pHRR increases from 136·to 219 kW·m^−2^, the TP decreases from 560 to 443 s, as the unit cell changes from G, D to S-P. The FGI increased from 0.2439 in G-type units to 0.4953 kW·m^−2^·s^−1^ in S-P type units with an increase of 50.77%. Because the Diamond TPMS structure is a symmetrical geometric arrangement consisting of a lattice structure composed of 14 nodes and 16 edges of the same length with a higher surface area compared with that of Schwartz [41], the porosity is crucial for its physical properties [42]. The diamond TPMS structure is characterized by a highly symmetric lattice geometry comprising 14 nodes interconnected by 16 equal-length edges. Owing to its larger specific surface area relative to the Schwarz TPMS structure [40], porosity plays a critical role in determining its physical properties [42]. While the Gyroid structure is characterized by the absence of straight lines or planar symmetry curves integrated into R3 [43], the absence of “through-hole” geometry in D-type structures leads to increased wall-to-fluid perturbation, which accelerates the fire growth compared to G-type structures [20]. Meanwhile, it reveals that S-P structures have the lowest flow resistance (∆p) and the highest integrated heat transfer coefficient (j/f) [44], thus enhancing the thermal conductivity [45] and the convection, resulting in the improved Nusselt number [46], leading to an increased FGI.

In summary, the fire growth rate exhibits irregular linear relationships with both the porosity and specific surface area, as shown in Figure 5, presenting a messy and irregular characteristic, and the porosity and specific surface are calculated using of AreanTopology 5.22.2 software, as shown in Table 4. It indicates that, within the present TPMS orthogonal dataset, the flame propagation does not exhibit a simple linear dependence on porosity or specific surface area alone and that it is primarily governed by the cell architecture. The irregular correlations between FGI and porosity/specific surface area in Figure 5 reveal that static geometric indices alone cannot fully characterize dynamic combustion heat feedback and oxygen diffusion. This originates from intrinsic differences in tortuosity, pore connectivity and curvature across three TPMS types, which control flow separation, turbulence and solid–fluid heat exchange, and finally determine flame spread speed. Gyroid with smooth curved channels weakens flame propagation; Diamond with complex nodes enhances fluid–wall interaction; Schwarz-P with low flow resistance and steep curvature gradients accelerates heat feedback and flame growth. Limited by this orthogonal screening design, separate contributions of these topological parameters are not decoupled here and will be analyzed via pore-scale simulation in future work. Therefore, the D-type TPMS PLA is mainly employed firstly to probe the effects of cell thickness, cell type, and cell size on flame propagation; it determines that they could all affect the fire diffusion rate. The orthogonal experiments provide an effective and efficient method to clarify the relationships between fire growth rate and cell structure.

Notably, the three TPMS samples possess comparable porosity and oxygen diffusion conditions, while their FGI varies dramatically. This phenomenon eliminates oxygen availability as the dominant factor governing flame propagation and indicates that the inherent heat transfer discrepancy induced by topological curvature and flow disturbance is the core reason for differentiated fire growth performance.

Even for samples with similar porosity (i.e., similar total combustible mass), the FGI values of three TPMS types differ significantly, demonstrating that mass fraction of solid PLA is not the dominant factor controlling flame propagation.

Table 4 presents the outcomes of the FGI range analysis for various TPMS. Here, K_n_ (*n* = 1, 2, 3) denotes the sum of FGI for TPMS porous structures at different levels under distinct factors, while k*_n_* (*n* = 1, 2, 3) signifies the average FGI of TPMS porous structures at different levels under different factors. Additionally, R represents the difference between the maximum and minimum at the level of each factor, serving to evaluate the influence of each factor on the FGI. The analysis findings indicate that the degree of influence of each factor on FGI follows the sequence: cell size > unit cell > thickness. Within factor A, k_1_ > k_2_ > k_3_, within factor B, k_1_ > k_2_ > k_3_, and within factor C, k_3_ > k_2_ > k_1_. It is essential to note that a reduction in FGI corresponds to a deceleration in flame growth; thus, prioritizing the case with the lowest FGI is imperative. At present, the optimal combination of levels is identified as A_3_B_3_C_1_. Test set 1 aligns with this combination, consistent with the intuitive analysis results in Section 3.1.

**Table 4:** Summary of flame propagation and porosity of samples.

Unit Cell	Unit Cell Size/mm	Thickness/mm	Porosity	pHRR /kW·m^−2^	Specific Surface Area/cm^2^·g^−1^	T_p_/s	FGI/kW·m^−2^·s^−1^
D	10	0.2	92.34%	54.32	71.4	541	0.100
D	10	0.4	84.75%	55.64	36.09	598	0.093
D	10	0.8	69.68%	56.06	18.3	620	0.09
D	10	1	62.43%	57.22	14.679	640	0.089
D	2	0.4	29.96%	82.41	42.56	494	0.166
D	5	0.4	69.76%	71.51	35.25	516	0.138
D	8	0.4	80.88%	62.43	35.934	644	0.096
G	10	0.4	87.65%	136.58	36.08	560	0.243
S-P	10	0.4	90.60%	219.45	35.95	443	0.495

All pHRR, *T_p_* and FGI data presented in this table are averaged from three repeated cone calorimeter tests, and the relative deviation of pHRR between replicates is less than 10%, which meets the repeatability requirement of BS ISO 5660-1:2015.

### 3.2. Orthogonal Experiment Analysis

Table 5 displays the results of the orthogonal tests. Therefore, considering the application’s objective, attention should be directed towards the group with the lowest FGI, namely, the first group. Table 6 presents the FGI range analysis of TPMS porous PLA, a smaller K value indicates a lower fire growth rate and slower fire growth. With the lowest k value of 0.371 and the highest R value of 0.157 among all samples, cell size is identified as the most influential factor governing fire growth. Consequently, it can be intuitively deduced that A_3_B_3_C_1_ represents the optimal combination among the nine groups examined.

All pHRR, *T_p_* and FGI data presented in this table are averaged from three repeated cone calorimeter tests, and the relative deviation of pHRR between replicates is less than 10%, which meets the repeatability requirement of BS ISO 5660-1:2015.

**Table 5:** Results of orthogonal test.

Unit Cell	Cell Size/mm	Thickness/mm	Porosity	pHRR /kW·m^−2^	Specific Surface Area/cm^2^·g^−1^	T_p_/s	FGI/kW·m^−2^·s^−1^
G	10	1.0	92.34%	54.00	14.71	610	0.089
S-P	8	0.4	84.75%	57.80	37.01	499	0.116
S-P	4	1.0	69.68%	66.79	9.45	463	0.144
D	10	0.4	62.43%	54.42	35.81	592	0.092
S-P	10	0.8	29.96%	55.11	18.78	565	0.098
D	8	1.0	69.76%	56.09	14.69	503	0.112
G	8	0.8	80.88%	55.90	18.18	513	0.109
D	4	0.8	87.65%	62.33	33.52	489	0.128
G	4	0.4	90.60%	61.21	35.62	494	0.124

All pHRR, *T_p_* and FGI data presented in this table are averaged from three repeated cone calorimeter tests, and the relative deviation of pHRR between replicates is less than 10%, which meets the repeatability requirement of BS ISO 5660-1:2015.

### 3.3. ANOVA of Fire Growth

The ANOVA results presented in Table 7 show F-values for thickness, cell size, and unit cell of 3.752, 189.812, and 31.811, respectively. It is noted that the ANOVA method alone does not adequately explain data fluctuations resulting from changes in factor levels and errors, necessitating further analysis.

In ANOVA, the critical F-values are F_0.05_(2,20) =3.49 for the significance level α = 0.05 and F_0.01_(2,20) =5.85 for *α* = 0.01. Comparing the calculated F-values with these critical thresholds, the F-value of wall thickness equals 3.752, which exceeds F_0.05_(2,20) but is lower than F_0.01_(2,20). This means thickness has a statistically significant effect on FGI only at *α* = 0.05. In contrast, the F-values of cell size (189.812) and unit cell (31.811) are both far greater than F_0.01_ (2,20) =5.85, revealing their highly significant impacts on FGI at the *α* = 0.01 level. The significance classification of each factor is summarized in Table 7.

**Table 7:** Analysis of variance for FGI.

Dependent Variable FGI	Class III Sum of Squares	Degree of Freedom	Mean Square	F	F_α_(2,20)	Significance Level
Thickness	0.002	2	0.001	3.752	0.05(3.49)	*
Cell size	0.121	2	0.061	189.812	0.01(5.85)	***
Unit cell	0.020	2	0.010	31.811	0.01(5.85)	***
Error	0.006	20	0.000			

Note: * Significant atα = 0.05; *** highly significant atα = 0.01.

### 3.4. Multiple Linear Regression

**Figure 6:**
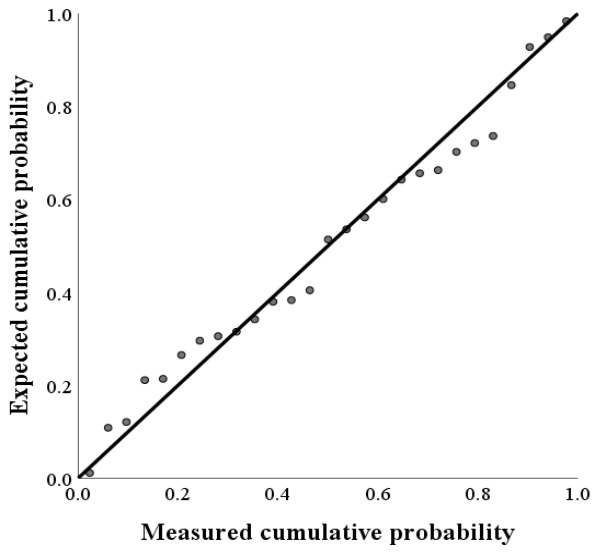
Regression standard residual normal p-p diagram.

**Figure 7:**
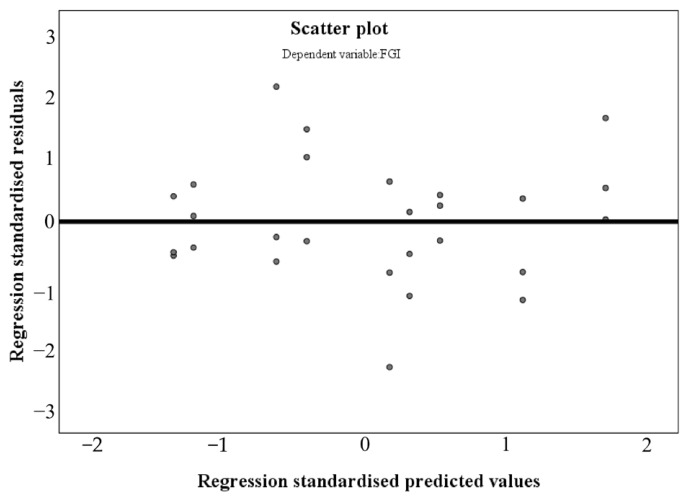
Scatter plot of standardized residuals.

The dependent variable was the FGI of TPMS porous PLA, and the independent variables were the thickness, cell size, and unit cell, which were analyzed through linear regression using SPSS software. Regression modeling was conducted on the full dataset of 27 independent observations (three replicates for each of the nine orthogonal combinations), which enables the model to capture both the main structural effects and inherent experimental scatter. The results of the linear regression analysis are summarized in Table 8, Table 9 and Table 10 and Figure 6 and Figure 7, showing the degree of fit of the regression model, respectively.

#### 3.4.1. Multiple Linear Regression Analysis

The experimental results were analyzed using multiple linear regression in SPSS with the Enter method. The three unit parameters were treated as independent variables, while FGI served as the dependent variable, allowing the collective predictive contribution of all independent variables to be evaluated.

Table 8 presents the evaluation metrics of the regression model, including the correlation coefficient (R), coefficient of determination (R^2^), adjusted R^2^, root mean square error (RMSE), mean estimation error (MAE), and Durbin–Wolston test statistics. The critical table for the correlation coefficient test is examined when *n* = 27(total individual experimental observations) and the significance level α = 0.01, yielding R_0.01_ = 0.479. Notably, regression analysis uses all 27 separate replicate measurements instead of group-averaged values to retain complete experimental variation information. The correlation coefficient, R = 0.975, indicates that the sample FGIs have strong correlations with the thicknesses, unit dimensions, and unit cell. Table 9 displays the ANOVA results of the regression model. The F-value is 144.837, and the *p*-value is 0.000, which is less than 0.01. This indicates that the model significantly explains the variance, and hence all the regression coefficients are significant, i.e., none of them are zero.

**Table 8:** Model summary.

Model	R	R^2^	Adjusted R^2^	RMSE	MAE	Durbin–Watson Test
1	0.975	0.950	0.943	0.0167	0.0130	2.019

**Table 9:** Analysis of variance.

Parameters	Sum of Squares	Degree of Freedom	Mean Square	F	Significance
Regression Residual	0.143	3	0.048	144.837	0.000
error	0.008	23	0.000	-	-
Total	0.150	26	-	-	-

In this research, an innovative FGI prediction model tailored for TPMS porous PLA is assessed for its accuracy using four widely recognized evaluation indicators: R-value, R^2^, RMSE, and MAE. A low RMSE value denotes a model’s precision in capturing the relationship between input and output variables. The lower values of RMSE and MAE highlight the model’s accuracy, predictive strength, and reliability in mirroring the experimental results. Equations (12)–(15) facilitate the computation of these metrics.

(12) $$R=\frac{{\left(N\sum_{i=1}^{N}x_{i}^{act}x_{i}^{pre}-\sum_{i=1}^{N}x_{i}^{act}\sum_{i=1}^{N}x_{i}^{pre}\right)}^{2}}{\left[N\sum_{i=1}^{N}x_{i}^{act^{2}}-{\left(\sum_{i=1}^{N}x_{i}^{act}\right)}^{2}\right]\left[N\sum_{i=1}^{N}x_{i}^{act^{2}}-{\left(\sum_{i=1}^{N}x_{i}^{pre}\right)}^{2}\right]}$$

(13) $$R^{2}=\frac{\sum_{i=1}^{N}(x_{i}^{act}-x^{-act}{)}^{2}-\sum_{i=1}^{N}(x_{i}^{act}-x_{i}^{pre}{)}^{2}}{\sum_{i=1}^{N}(x_{i}^{act}-x^{-act}{)}^{2}}$$

(14) $$RMSE=\sqrt{\frac{\sum_{i=1}^{N}(x_{i}^{act}-x_{i}^{pre}{)}^{2}}{n}}$$

(15) $$MAE=\frac{\sum_{i=1}^{n}\left|x_{i}^{act}-x_{i}^{pre}\right|}{n}$$

where *N* represents the number of samples in the data; x_i_^act^ and x_i_^pre^ denote the measured and predicted values of FGI, respectively. In this study, the model has an R-value of 0.975. The R^2^ of 0.950 indicates that the thickness, cell size, and unit cell explain 95% of the variation in FGI, whereas the RMSE and MAE are 0.0167 and 0.0130, respectively.

In conclusion, the FGI of the samples shows a clear linear relationship with thickness, cell size, and unit cell, and the regression coefficient is non-zero, allowing a linear regression equation to be established.

#### 3.4.2. Establishment of Multiple Linear Regression Equations

Table 10 presents a summary of the coefficients for the regression model, including the unstandardized regression coefficients (B-value column), standardized regression coefficients (Beta-value column), t-values, and probability of significance values (*p*-values) used for regression coefficient significance tests, along with statistics used to diagnose the covariance tolerance and variance inflation factor (VIF). The larger the absolute value of the standardized regression coefficient, the more significant the effect of the independent variable on the dependent variable.

Analyzing the regression coefficients in Table 10, it is evident that the Sig-t-values for cell size and unit cell are less than the 0.001 level of significance. In contrast, the Sig-t-value for thickness exceeds 0.01, suggesting that the effect of thickness on the FGI is not significant. However, the impact of the size and type on FGI is more substantial than the thickness. Meanwhile, a low tolerance value nearly approaching zero typically indicates potential linear dependency among variables, whereas a VIF exceeding 10 points to significant multicollinearity issues. However, the analysis shows tolerance values for all considered variables—thickness, cell size, and unit cell—as 1, with corresponding VIFs also recorded at 1, thus demonstrating no evident multicollinearity.

**Table 10:** Regression coefficient.

	Unstandardized Coefficient		Standardization Coefficient	t	Significance	Covariance Statistics	
	B	Standard Error	Beta		*p*	Tolerance	VIF
(Constant)	0.526	0.015		34.639	0.000		
Thickness	0.011	0.004	0.122	2.603	0.016	1.000	1.000
Cell size	−0.082	0.004	−0.897	−19.194	0.000	1.000	1.000
Unit cell	0.033	0.004	0.360	7.703	0.000	1.000	1.000

The regression equation, primarily employing unstandardized B coefficients for predicting test sample outcomes, contrasts with the use of standardized Beta coefficients, which cater more to theoretical explorations. Consequently, the applied regression equation for estimating the FGI predominantly utilizes the B-values for practical purposes. The unstandardized regression equation can be formulated as follows:

(16) $$f_{FGI}=0.1315+0.00275W_{Thickness}-0.0205W_{Size}+0.00825W_{Type}(R=0.975)$$

where the f_FGI_ represents the fire growth of TPMS porous PLA in kW·m^−2^·s^−1^, W_Thickness_ is denoted the thickness in mm, W_Size_ signifies the cell size in mm, and W_Type_ refers to the unit cell, respectively.

Previous studies have shown that the G and D structures are well suited for applications involving high strain, whereas the Schwarz structure is more appropriate for low-stress conditions [47]. In addition, the Schwarz architecture has been reported to exhibit the highest gauge factor among these TPMS configurations [48]. Our study has compared and analyzed the fire growth of different cell TPMS structures from the perspective of combustion heat release, establishing the regression equation on its fire growth.

#### 3.4.3. Tests of Multiple Linear Regression Equations

Table 11 displays the residual statistics, where the highest standardized residual value is under 3, confirming that these values are acceptable. Moreover, the reduced standard deviation of the residuals indicates that the regression model adequately captures the relationship between thickness, size, and type, and the FGI is consistent with the experimental observations.

The normality of the 27 data sets is evaluated through a standardized residual normality probability–probability plot in Figure 6. The cumulative probability distribution of the standardized residual values forms a straight line at a 45-degree angle from the lower left to the upper right, which indicates that the sample observations meet the normality assumption. Furthermore, the analysis presented in Figure 7 demonstrates that the cumulative probability point distribution of the standardized residuals for the FGI closely follows the theoretical trend line, suggesting that the data approximate a normal distribution.

In Figure 7, a scatter plot is presented, depicting standardized residuals versus standardized predicted values of the FGI, aimed at assessing the normality and variance uniformity. The scatterplot reveals symmetrically centered points at 0, with a random horizontal distribution, and all data points fall within ±2 standard deviations. These findings suggest that the sample observations meet the assumptions of normality and variance uniformity. It indicates a correct linear relationship combining its FGI with the thickness, size, and type, with overall positive outcomes and no outliers detected, further supporting the assumptions of normality and variance uniformity. To derive the predicted values of the FGI for the samples, the raw data of the three independent variables were inputted into the regression Equation (16). Overall, the regression equations performed satisfactorily.

The optimal A_3_B_3_C_1_ architecture refers to a Gyroid lattice with a 1.0 mm wall thickness and a 10 mm cell size. The maximum wall thickness of 1.0 mm increases the structural relative density, which improves compressive stiffness and yield strength to offset the mechanical degradation induced by large cell sizes [49]. The Gyroid topology exhibits uniform stress distribution and experiences stable layer-by-layer collapse under compression over a broad plastic deformation range, offering superior energy absorption compared with Diamond and Schwarz-P structures [50].

Distinct performance trade-offs exist between structural parameters: reducing cell size can boost stiffness and volumetric energy absorption [51], yet it markedly increases FGI and accelerates flame spread, as demonstrated in our orthogonal experiments. Thinner walls degrade mechanical performance and simultaneously facilitate flame propagation. The A_3_B_3_C_1_ design strikes a balanced compromise between fire resistance, compressive stiffness, yield strength and energy absorption, as it moderately sacrifices volumetric mechanical performance to achieve the lowest fire growth risk.

Finally, porous structures can significantly affect the combustion rate of materials by altering their cell size and thickness, but the mechanical properties should also be considered in practical applications. The TPMS grid configuration is characterized by an orthogonal architecture that often exhibits anisotropic mechanical behavior, resulting in stress concentrations at node intersections and inefficient load transfer along diagonal directions. When subjected to bending or impact loading, localized buckling or premature failure may occur at these junctions due to insufficient diagonal reinforcement [52]. Increasing the structure thickness has been reported to enhance both compressive strength and elastic modulus [53]. This article analyzes the influence of different cell structures on the fire growth rate from the perspective of flame propagation only. The proposed regression equation favors designing thermal management materials, but it requires further research to probe the detailed combustion mechanism in the porous polylactic acid.

## 4. Conclusions

Single-factor experiments eliminate porosity and oxygen availability as dominant control factors; topological heat transfer capacity is the primary driver. Notably, all heat release data were normalized by specimen mass to eliminate the influence of different combustible mass induced by varying wall thickness and cell size. This study systematically investigates how TPMS porous structure parameters influence the fire growth of 3D-printed PLA. Three TPMS models were produced using 3D printing technology and subjected to controlled combustion using an orthogonal experimental design. To the best of the authors’ knowledge, this study is the first to explore the effects of different TPMS types on the fire growth of additively manufactured polylactic acid, focusing on thickness and cell size, using experimental methods of the cone calorimeter and 3D printer. The results demonstrated that fire growth is primarily governed by TPMS architecture rather than by porosity or specific surface area alone. And the orthogonal experiment reveals that the influence on the FGI follows the order: cell size > unit cell > thickness. Furthermore, a multivariate regression model was developed for predicting the FGI of TPMS porous polylactic acid, demonstrating high prediction accuracy and a small residual standard deviation. We also acknowledge that the high-precision regression model (R^2^ = 0.95) does not separate the independent effects of tortuosity, connectivity and curvature, as all topological influences are lumped into cell type due to the orthogonal test limitation. Future work will combine X-CT reconstruction, pore-scale simulation and supplementary TGA/DSC thermal analysis to extract multi-topological descriptors and matrix thermal degradation parameters, establish a coupled fire growth prediction model, and reveal how TPMS topology mediates polymer flame propagation via heat and mass transfer.

## Figures and Tables

**Table 1:** Experimental factors and levels.

Horizontal Factor	Thickness/mm	Cell Size/mm	Unit Cell
1	0.4	4	Gyroid
2	0.8	8	Diamond
3	1.0	10	Schwarz-P

**Table 2:** The L9 (3^3^) orthogonal experimental design.

Serial Number	Thickness/mm	Cell Size/mm	Unit Cell
1	1.0	10	1
2	0.4	8	3
3	1.0	4	3
4	0.4	10	2
5	0.8	10	3
6	1.0	8	2
7	0.8	8	1
8	0.8	4	2
9	0.4	4	1

**Table 3:** Univariate experiment design.

Variable	Thickness/mm	Cell Size/mm	Unit Cell
Wall thickness	X	10	D
Cell size	0.4	X	D
Cell type	0.4	10	X

**Table 6:** FGI range analysis of TPMS porous PLA.

Serial Number	Thickness/mm	Cell Size/mm	Unit Cell	FGI/kW·m^−2^·s^−1^
1	1.0	10	G	0.089
2	0.4	8	S-P	0.116
3	1.0	4	S-P	0.144
4	0.4	10	D	0.092
5	0.8	10	S-P	0.098
6	1.0	8	D	0.112
7	0.8	8	G	0.109
8	0.8	4	D	0.128
9	0.4	4	G	0.124
K_1_	1.378	1.583	1.286	—
K_2_	1.337	1.346	1.325	—
K_3_	1.327	1.113	1.431	—
k_1_	0.459	0.528	0.429	—
k_2_	0.446	0.449	0.442	—
k_3_	0.442	0.371	0.477	—
R	0.017	0.157	0.048	—

**Table 11:** Residual statistics.

Data	Minimum Value	Maximum Value	Average Value	Standard Deviation
Predicted value	0.34672	0.57639	0.45044	0.074048
Residual error	−0.041556	0.038611	0.000000	0.017036
Standard predicted value	−1.401	1.701	0.000	1.000
Standard residual	−2.294	2.132	0.000	0.941

## Data Availability

The raw data supporting the conclusions of this article will be made available by the authors on request.
